# Scanning Thermal Microscopy Method for Self-Heating in Nonlinear Devices and Application to Filamentary Resistive Random-Access Memory

**DOI:** 10.1021/acsnano.4c12784

**Published:** 2025-01-29

**Authors:** Nele Harnack, Sophie Rodehutskors, Bernd Gotsmann

**Affiliations:** IBM Research Europe − Zurich, 8803 Rüschlikon, Switzerland

**Keywords:** scanning thermal microscopy, nanoscale thermometry, calibrationless, self-heating, quantitative, nonequilibrium, resistive RAM

## Abstract

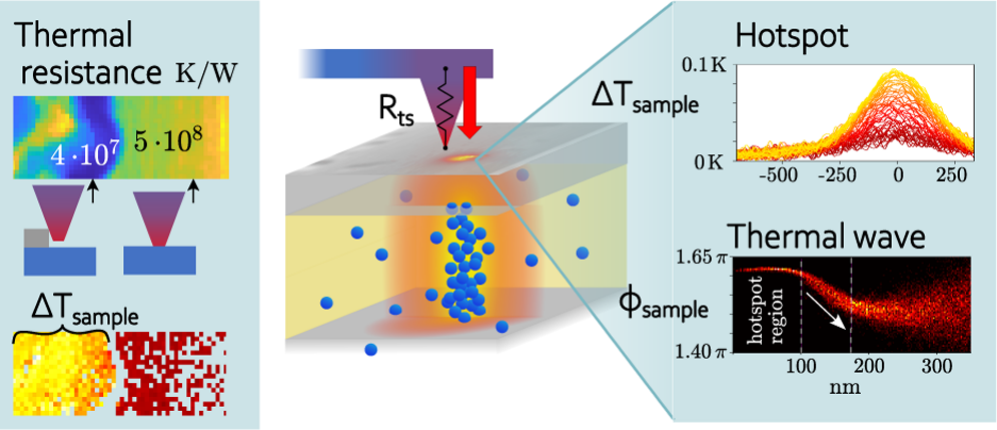

Devices with a highly nonlinear resistance-voltage relationship are candidates for neuromorphic computing, which can be achieved by highly temperature dependent processes like ion migration. To explore the thermal properties of such devices, Scanning Thermal Microscopy (SThM) can be employed. However, due to the nonlinearity, the high resolution and quantitative method of AC-modulated SThM cannot readily be used. To this end, an extended nonequilibrium scheme for temperature measurement using SThM is proposed, with which the self-heating of nonlinear devices is studied without the need for calibrating the tip–sample contact for a specific material combination, geometry or roughness. Both a DC and an AC voltage are applied to the device, triggering a periodic temperature rise, which enables the simultaneous calculation of the tip–sample thermal resistance and the device temperature rise. The method is applied to HfO_2_-based RRAM devices, in which the kinetic processes of filamentary switching are governed by temperature. We image temperature and propagation of thermal waves and extract properties like the number of current filaments, thermal confinement and thermal cross-talk.

## 1 Introduction

The self-heating of integrated microelectronic devices during their operation has received considerable attention. On the one hand, the high current densities and subsequent temperature increase in devices and leads are known to be the main cause for their failure.^1^ On the other hand, the temperature rise can also be exploited to create device functionality. In either case, it is of high importance to understand and control self-heating in scaled devices, but several factors make it difficult to both predict and measure device properties: the multiscale nature of heat transport, different self-heating effects, and different materials and material interfaces, which are often poorly characterized in their nanoscale thermal properties.

An important example are RRAM devices for neuromorphic computing architectures, which often use self-heating to change and store the resistive state.^2^ The mechanism consists of the movement of ions in the applied electric field in a strongly temperature-dependent kinetic process. During switching to the low-resistive state, the temperature is increased by self-heating, leading to a self-acceleration of the process as the resistance decreases and the Joule heating increases.^3^ Given that the nonlinear thermal response is essential for device operation, it is surprising how few studies exist that access the temperature of such devices,^4−6^ and how little is still known about their thermal properties, making it crucial to create high resolution and quantitative analysis methods.

Many methods of device thermometry with imaging capability have been applied to characterize self-heated devices, such as a range of scanning probe-based^7^ and optical methods like Raman thermography.^8^ Scanning Thermal Microscopy^9,10^ is particularly versatile as it can be applied to a large variety of samples under realistic operating conditions and routinely delivers high resolution.^11^ However, as we will discuss below, one issue of SThM is the difficulty of extracting quantitative information from the recorded electrical signals^7^ caused by the complex thermal coupling through the tip–sample contact.^12^ Methods to mitigate this issue include the null-point method^13^ or careful simulation of the tip–surface thermal contact.^9,12^ Another versatile SThM method modulates the voltage (or current) applied to a device at a given frequency and uses phase sensitive detection of the thermal sensor at its harmonics to extract quantitative information, which has been used in SThM and thermoreflectance imaging.^14^ However, while applicability to linear devices has been shown,^12,15^ such AC modulation methods cannot be applied directly to nonlinear electrical devices.

In this paper, we demonstrate high-resolution imaging of hotspots to characterize current filaments in RRAM devices based on HfO_2_ layers sandwiched between TiN metal electrodes. This is enabled by an extension of the SThM methodology toward nonlinear devices, obtaining a quantitative temperature in a single measurement without additional calibration. The method is sensitive enough to image self-heating effects through the cooling electrodes and enables extraction of important thermal dissipation characteristics.

The paper is organized as follows. We first derive the equations for the new method, discussing assumptions, strengths and limitations. To demonstrate that the method is quantitative, we use metal thin film resistors, of which the self-heating temperature can be determined independently. We then move on to motivate the study of HfO_2_-based RRAM devices and show the extraction of important properties through the application of SThM. We continue with more common questions on the temperature and size of the current filament during operation before turning to time-dependent properties, which can now be accessed using the demonstrated method.

## 2 Results

### 2.1 SThM Technique for Linear and Nonlinear Devices

#### 2.1.1 Equilibrium Thermometry and Nonequilibrium Thermometry Using SThM

**Figure 1:**
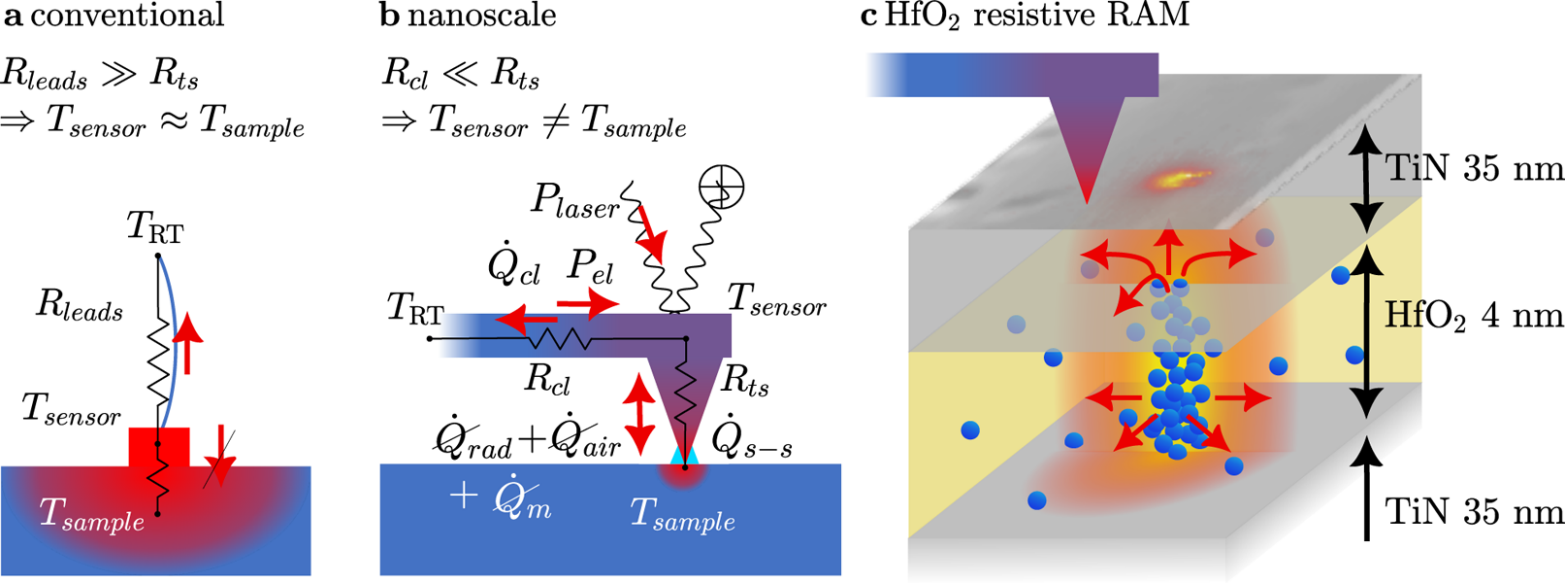
(a) In conventional thermometry at large scales, the sensor is in thermal equilibrium with the sample to be measured, resulting from the thermal resistance of the sensor leads being small in comparison to the thermal resistance between sensor and sample. As a result, there is no heat flow between sample and sensor and the temperature of the sample can be read directly from the sensor temperature. (b) In contact-based high-resolution techniques, the thermal resistance of the cantilever legs *R*_cl_ is lower than the resistance between tip and sample *R*_ts_ in order to minimize the influence of the measurement on the sample temperature. To quantify a sample temperature correctly in nonequilibrium SThM, different sources of heat flux *Q̇* need to be considered. Vacuum-based measurements enable avoiding contributions from air conduction and minimize water meniscus. (c) HfO_2_-based RRAM device with conductive filament consisting of oxygen vacancies (indicated by blue spheres). Heat is dissipated from the filament in radial and axial direction, of which the resulting temperature at the top electrode is observable.

In Figure 1a,b, conventional contact-based thermometry is compared to nanoscale temperature measurements. Depicted is the main difficulty in SThM: high resolution techniques do not operate in equilibrium of sensor and sample. The heat flux between sample and sensor is controlled by the thermal resistances between the sensor and the sample, *R*_ts_. While the tip-cantilever thermal resistance remains constant, the tip–sample thermal resistance at the contact between probe and sample can be highly variable during the scan and cannot be assumed to be constant.^11^ It is affected by variations of contact area, given by device topography, surface roughness, the material combination’s elasticity, the thermal conductivity of the sample and the thermal boundary resistance at the tip–sample interface. While features which have a similar size or are larger than the characteristic size of the tip–sample contact can be analyzed using a calibration approach or using simulations, smaller features have to be addressed differently.^5^

One proposed method is the use of a dual-scan mode,^12^ i.e., the combination of active and passive mode SThM to extract both tip–sample thermal resistance and temperature in a single measurement instead of two.^16^ This is conceptually similar to varying the probe temperature to extrapolate to the null-point,^13^ as both methods are able to account for the variation in tip–sample thermal resistance. It has been successful in removing artifacts from measurements of various devices while maintaining a very high resolution.^12,15^ However, the methods proposed so far are only suitable for linear resistors in which the self-heating can easily be interpreted. For example, in a linear resistor modulated with a sinusoidal current or voltage at frequency *f*, the self-heating power due to Joule heating will be modulated at 2*f*, while Peltier effects can be observed at 1*f*.^17^ If, in addition, the thermal resistance (coupling the device to its environment) is linear and the modulation small and sufficiently slow, the local temperature rise Δ*T*(*x*, *y*) will also be modulated with 2*f*. This relation (Δ*T* ∝ 2*f*) translates readily to resistor networks and can account for heat spreading around the heat source in a device. When the resistor is nonlinear, however, i.e., the current is no longer proportional to voltage, the simplified assumption cannot be made. Additionally, the investigation of devices at different offset voltages (also often referred to as ‘DC bias’) can reveal their characteristics at realistic operating conditions, opening up the possibility of characterizing volatile states.

Here, we explore ways to use modulation techniques and phase-sensitive detection at different applied offset voltages, with the goal of extracting quantitative information from the temperature fields. To this end, a sensing and data analysis scheme for devices at constant offset voltage was developed based on the dual-scan method.^12^ The device is biased by a voltage with a constant offset (DC voltage) and an oscillating voltage (AC voltage). This induces an oscillating temperature in the device, which causes an oscillating heat flow between sample and probe and thus an oscillating resistance of the probe’s integrated thermistor. As in the original dual-scan mode, the tip–sample thermal resistance is not assumed to be constant, but will be calculated independently for each image pixel. The advantage of this modified dual-scan SThM method in comparison to the null-point method or modulating the heating current of the tip does not lie in simplifying assumptions or increasing elegance. It is, however, very easy to practically implement and in our tests shows better sensitivity and fewer artifacts. In the small signal limit, all methods coincide in their basic assumptions.

#### 2.1.2 Derivation of Governing Equations

In this scheme, a device is electrically driven through modulation of a bias voltage (or, equivalently, a current) at modulation frequency *f*, including both AC and DC components:
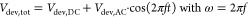
1

The resulting Joule heating induces a temperature rise of the sample, which is also periodic:
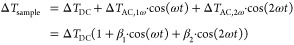
2plus higher order terms. Here, β_1_ and β_2_ are introduced, and, as will be discussed below, can be determined independently. This leaves us with one unknown for the device, Δ*T*_DC_, that is to be determined using this method for each pixel.

To analyze the relation between device temperature rise and operating conditions, one must choose the modulation frequency ω to be sufficiently low. In that case, the device maintains a steady state, i.e., a constant temperature within a time span that the applied voltage is almost constant. In other words, the modulation frequency should stay well below the device’s cutoff frequency. Experimentally, this frequency can be determined by measuring the device’s frequency response in the third harmonic.^18^ It follows that the device temperature rise is proportional to the power dissipated in the device, which allows us to compute the β-values. Additionally, the temperature rise in the device is assumed to be small, such that the thermal conductance of the device material and the thermal resistance of the tip–surface contact can be assumed independent of temperature and constant within one pixel.

Next, the sensor’s temperature out of contact and in contact with the sample are described. Out of contact with the sample, the sensor has a temperature that is elevated by Δ*T*_sen,0_ above room temperature *T*_RT_, caused by self-heating through the voltage *V*_0_ applied to it via a Wheatstone-bridge. When the probe is brought into contact with the sample, its temperature changes due to the interaction with the sample. This induced temperature change is described by Δ*T*_sen_, which can have DC and/or AC contributions, depending on the temperature changes that occur in the sample. The total temperature in the cantilever is then
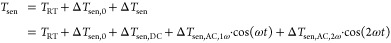
3

The sensor temperature rise is calculated via its calibrated temperature-dependent resistance and for small temperature changes can be linearized around the working point *R*_0_ = *R*(Δ*T*_sen,0_) to
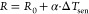
4

The resistance change is detected through the voltage drop over the cantilever
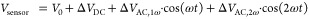
5

In a first approximation, the current can be treated as constant, because the periodic resistance changes Δ*R*_AC_ are small in comparison to the constant resistance of the heater Δ*R*_DC_ and its series resistance *R*_series_:
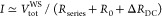
6where  is the voltage applied to the Wheatstone bridge. Then the temperature changes can be calculated directly from the corresponding constant or periodic voltage change Δ*T*_DC_ = Δ*V*_DC_/(*I* · α). This assumption is written for simplicity and ease of understanding. For a more detailed treatment without assuming a constant current, which in many cases should be chosen over the simple approach, see the Methods.

To relate the local temperature change in the sample to the measured change in cantilever voltage, the continuity equation (energy conservation) of the cantilever is used, as depicted in Figure 1.

First, for the tip out of contact with the sample, all the power dissipated in the sensor is conducted as heat along the cantilever legs (see Figure 1):
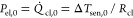
7

We neglect radiation or air conduction in our vacuum setup. If present, these can be taken into account in a single *Q̇*_cl_ and eliminated in the measurement through the same calibration step used now. Then, for the in-contact case
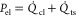
8and
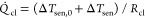
9

Now the tip–sample contact provides an additional conduction channel, and the heat flux *Q̇*_ts_ can be described as
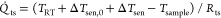
10

The electrical power of the heater can be calculated as *P*_el,0_ = *V*_0_ · *I* in the out-of-contact case and *P*_el_ = (*V*_0_ + Δ*V*) · *I* in the in-contact case.

Plugging the terms for the device, eq 2, and the sensor temperature, eq 3, the sensor resistance and voltage, eqs 4, 5, the terms for the cantilever out of contact, eq 7, and the heat-flows in contact, eqs 9, 10 into the energy equation in contact, eq 8, then separating the equation for the constant and first and second harmonic contributions *n* = 1, 2, yields a system of three equations:
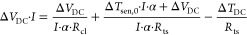
11
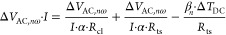
12

The remaining unknown variables are the sample’s temperature rise Δ*T*_DC_ and the tip–sample thermal resistance *R*_ts_. Equations 11 and 12, together with 6, are used to solve for the unknown local sample temperature Δ*T*_DC_ and the tip–sample thermal resistance *R*_ts_, to finally infer the device’s constant temperature rise 
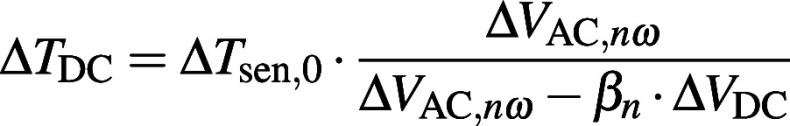
13

The harmonic *n* = 1, 2 can be chosen according to which signal has the larger signal-to-noise ratio, and the periodic temperature rise in the device Δ*T*_AC,*n*ω_ can then be calculated using eqs 16 or 17 respectively. The tip–sample thermal resistance is calculated as
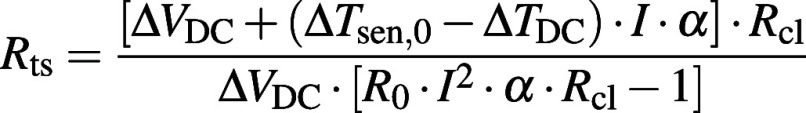
14with *R*_cl_ = Δ*T*_sen,0_ /(*V*_0_ · *I*).

In the case of no offset voltage applied to the device, *V*_dev,DC_ = 0, we obtain β_1_ = 0 and β_2_ = 1, such that eq 13 simplifies to Δ*T*_DC_ = *T*_sen,0_ · Δ*V*_AC,2ω_ /(Δ*V*_AC,2ω_ – Δ*V*_DC_), as was found by Menges et al.^12^

We note that the tip–sample thermal resistance depends on several factors, including the heat-spreading into the sample, which is governed by the thermal exchange radius and sample thermal conductivity.^19^ Since *R*_ts_ is extracted from measurement data, variations in these factors are implicitly taken into account. Determining the separate contributions of *R*_ts_ to obtain e.g., sample thermal conductivity is a common objective of SThM,^7^ but is not the aim of this method and not needed for determining the temperature.

#### 2.1.3 Verification Using a Metal Thin-Film Heater

To verify that the proposed method is capable of measuring quantitative temperature rises, a metal thin-film heater device is imaged using this dual-scan SThM method. The temperature of the device is determined independently (see Supporting Information). The device consists of a rectangular shaped Pt thin film of 50 μm length, 400 nm width and 120 nm thickness with four contact pads deposited on a film of 80 nm SiO_2_ on a Si substrate. When operated as a self-heated device, its temperature rise in dependence of the applied current is calculated from the resistance change . Here, the calibrated temperature coefficient of resistance of the Pt film is TCR = 1.9 × 10^–3^ K^–1^.

In a first step, the SThM sensor needs to be calibrated. As was identified by Spieser et al.,^20^ the available fixed-point calibration can yield an error of up to ∼20% for the empirical law describing the temperature of the sensor in relation to the electrical power of the cantilever, with an estimated maximum of +10% error due to deviation of the specific cantilever used in this measurement. To improve the calibration, we use the already-verified dual-scan method without offset,^12^ i.e., only applying an AC voltage to the sample. A set of scans with different amplitudes *V*_dev,AC_ and no offset *V*_dev,DC_ = 0 applied to the device were taken, and the temperature was found as described above. By comparing the expected with the measured temperature, the cantilever was calibrated, obtaining a working point of Δ*T*_sen,0_ = 183 K and α = 1.25 Ω/K.

In a second step, we turn to the case of adding a large DC bias to the sample. The corresponding β-values are easily determined. For small ω, we calculate:
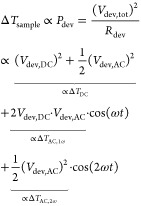
15which shows that the total temperature rise consists of a constant temperature rise and a periodic temperature rise in the first and second harmonic.

Next, we calculate the ratios between the temperature rises
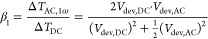
16and
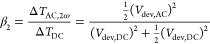
17

**Figure 2:**
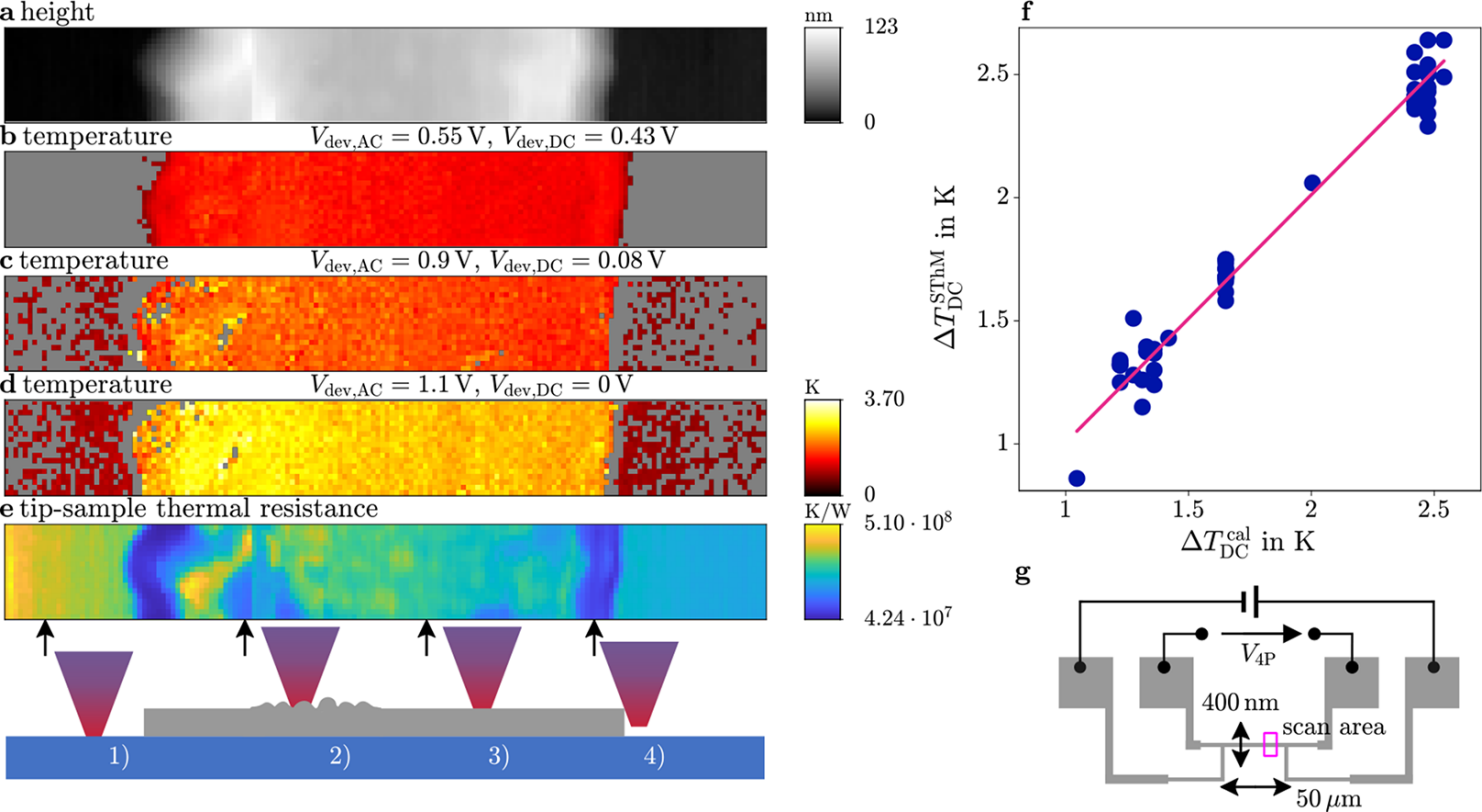
Verification of the temperature measured in SThM. (a) Height measurement of line heater device. (b–d) SThM temperature rise distribution at different DC and AC voltages applied. The temperature rise in each pixel (100 × 800 per image, with 5 nm resolution) is calculated from the equations for the nonconstant current case, and the average temperature rise is calculated by taking the average over the device area in each image. (e) Tip–sample thermal resistance as measured in SThM, in different tip–sample contact cases: (1) sample region of low conductivity (substrate) (2) defective region of Pt film with large surface roughness (3) sample region Pt film of high conductivity (4) edge of Pt film with large contact area. (f) Averaged DC temperature rise measured in SThM, averaged per scan over the device area, plotted against the calibrated device temperature in blue and a linear least-squares fit *y* = 1.0061 · *x* in pink. (g) Device schematic with the scan area indicated by the pink box.

Scanning Thermal Microscopy measurements were conducted for a range of device operating conditions that would result in a range of expected DC temperature rises  from 1 to 2.5 K which were then compared to the SThM-measured temperature rises . Three examples of this set of measurements are shown in Figure 2, where the temperature images in b-d illustrate the rise in temperature in the phase-locked pixels when raising the applied voltage. A detailed description of this plotting procedure is given in the Supporting Information. We calculated the average temperature rise over the device area, as indicated in Figure 2g by the pink box. The result of the measurement series is plotted in Figure 2f, where each scan is represented by a single data point of its average DC-temperature rise. A line *y* = *m* · *x* was fit to all data points using a least-squares error function, yielding a slope *m* = 1.0061 with goodness of fit *R*^2^ = 0.97. This shows excellent agreement between the measured and calibrated device temperature using our new method. This result is a verification of the equations derived above, the experimental implementation, and, importantly, the underlying assumptions.

### 2.2 Self-Heating of RRAM Devices

#### 2.2.1 HfO_2_-Based RRAM Devices: Open Questions

Hafnium Oxide (HfO_2_)-based memristors have been studied as candidates for neuromorphic computing devices.^2^ To operate devices in a bipolar way, applying a voltage *V*_SET_ to the device is used to switch the device into the low resistive state (LRS), and a voltage *V*_RESET_ of opposite polarity can be applied to reset it to the high resistive state (HRS). The fundamental mechanisms, however, are still being explored. Nanoionic processes, i.e., the migration of ions/oxygen vacancies, are used to explain the switching and failure mechanisms in HfO_2_-based and other RRAM devices.^2^ For example during SET switching, the migration of ions is dependent on temperature. This creates a positive feedback loop via the increased Joule heating with decreasing resistance, and accelerates the closing of the ruptured filament’s gap to retain the LRS. The dependence on temperature is often described and parametrized as a kinetic process, which explains the dependence of retention time, switching time and filament size on compliance current to a satisfactory level.^21−23^

However, the actual magnitude of the temperature inside devices is often unknown. Moreover, the heat dissipation in highly integrated crossbar arrays needs to be carefully engineered to avoid thermal crosstalk between neighboring devices, which lower retention time and influence the robustness of operation parameters. However, the actual size of the heat source, which is not necessarily equal to the filament, its temperature, which is not necessarily uniform, and the decay of temperature in the different layers depend on many factors. They are determined by the geometry, mode of transport (diffusive/ballistic/quantum), thermal conductances, and thermal boundary resistances of the surrounding materials and interfaces. Therefore, a deeper understanding of the thermal processes inside RRAM, lead by highly resolved thermometry, is essential for reliability and ultimate scaling goals.

**Figure 3:**
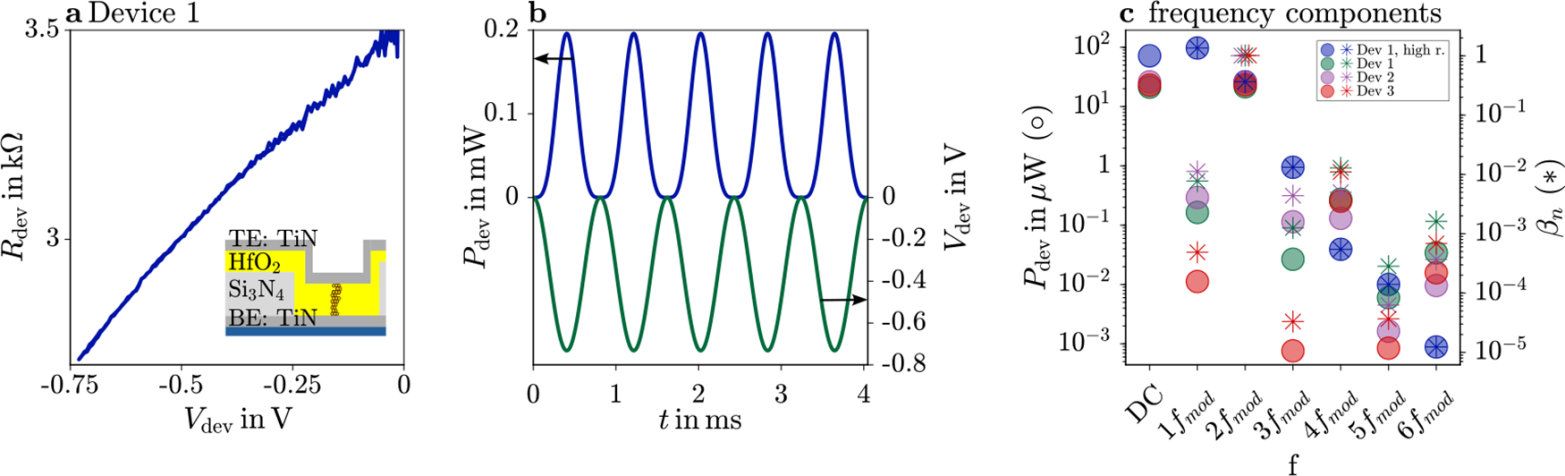
Resistance-Voltage characteristic of device 1 (a) and device voltage and power over time of device 1 in high-resolution scan (b). Power frequency components, marked with filled circles, and resulting β_*n*_, marked as asterisks, of device 1 for high (blue) and low (green) resolution images and device 2 (lilac) and 3 (red) (c).

To address these open questions using the method introduced here, we studied HfO_2_-based RRAM fabricated and electrically characterized by Stellari et al.^24^ The device design is schematically depicted in Figures 1c and 3a. It consists of an active layer of 4 nm HfO_2_ with a 35 nm TiN bottom- and top electrode on a bulk Si substrate. The separation between the electrodes is achieved by a Si_3_N_4_ interlayer, in which an opening of 4 μm × 4 μm defines the device area. During filament formation a compliance of 100 μA was set.^24^

#### 2.2.2 Dual Scan SThM Method Applied to Nonlinear Devices

In the case of a nonlinear device *R*_dev_ ≠ const with Joule heating, a numerical solution for β_1_ and β_2_ can be calculated using the resistance-voltage characteristics. Subsequently, eqs 13 and 14 can be used to infer the temperature and the tip–sample thermal resistance as before.

To find the temperature ratios β_1_ and β_2_, we need the temperature rise over time, which is again assumed to be proportional to the dissipated heat over time, Δ*T*(*t*) ∝ *P*_dev_(*t*). We start with the device resistance-voltage relationship as shown in Figure 3a, and calculate the dissipated device power as a function of voltage *P*_dev_(*V*_dev,tot_). Then we insert the time dependence, eq 12, to calculate *P*_dev_(*t*), as shown in Figure 3b. Third, a Discrete-Fourier-Transform is applied to *P*_dev_(*t*) to determine the contributions of the harmonics of the modulation frequency, the result of which is shown in Figure 3c with filled circles. Next, the amplitudes of the constant part *P*_dev,DC_ = 71 μW and the oscillations at the modulation frequency *P*_dev,AC1ω_ = 97 μW and double the modulation frequency *P*_dev,AC2ω_ = 27 μW are extracted. The β are calculated to β_1_ = *P*_dev,AC1ω_/*P*_dev,DC_ = 1.35 and β_2_ = *P*_dev,AC2ω_/*P*_dev,DC_ = 0.37. The calculated β_*n*_ are shown in Figure 3c as asterisks for all higher harmonics. The values rapidly decrease with increasing *n* and have high values for *n* = 2, justifying basing the analysis on this harmonic. Finally, eq 13 is used to infer the local constant device temperature rise Δ*T*_DC_ from the measured Δ*V*_AC,2ω_ and Δ*V*_DC_.

## 3 Discussion

**Figure 4:**
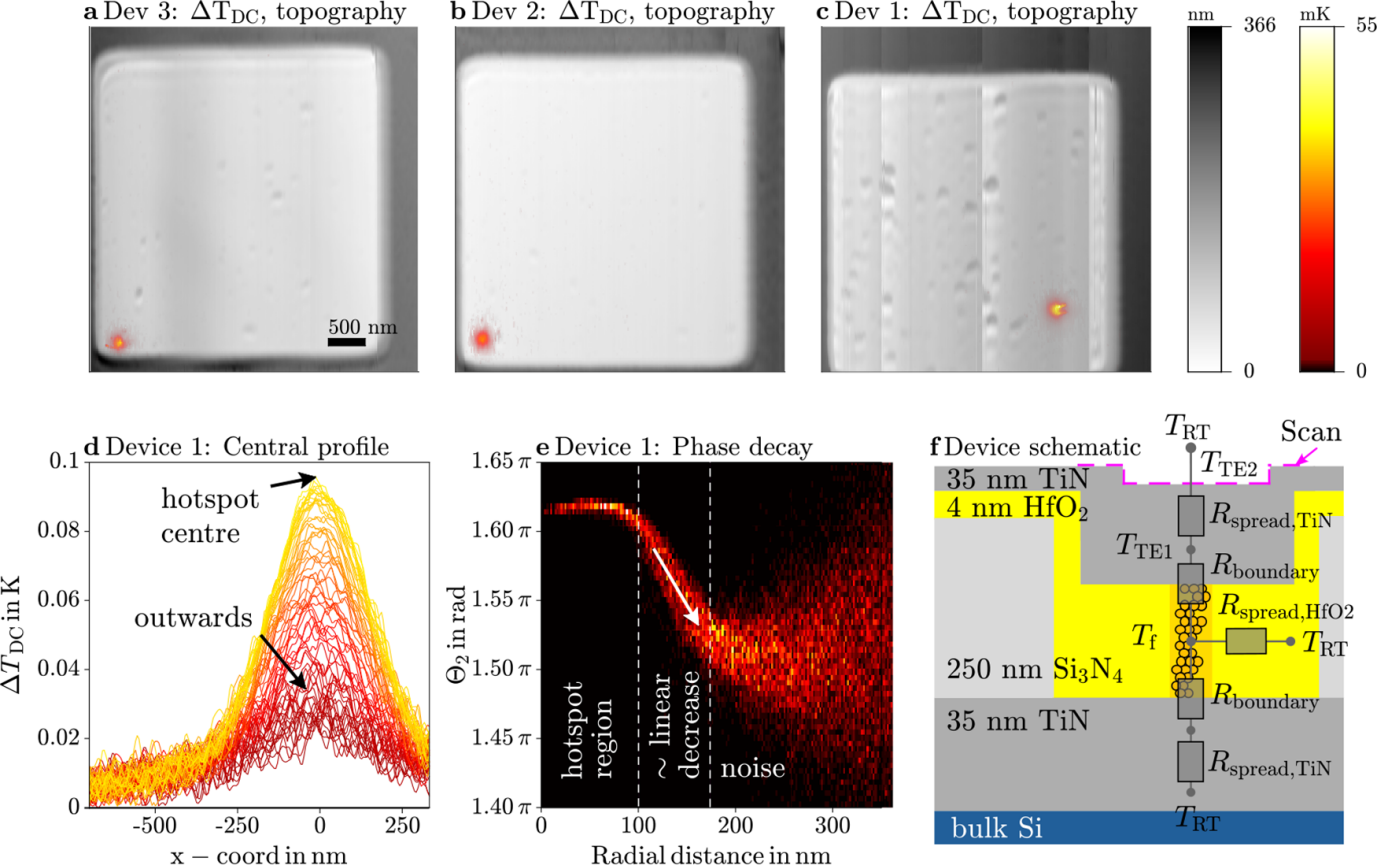
SThM temperature images of three different RRAM devices without offset applied (a–c). DC temperature profiles through center of hotspot (d) and decrease of phase with radial distance to hotspot center (e) from high resolution scan of device 1 with offset applied. The full data and detailed analysis including the detection of an artifacts in the hotspot region of this scan without offset applied is presented in the Supporting Information. Device schematic with thermal resistances (f).

The resulting DC temperature rises are shown in Figure 4 for three different devices. In each of the devices, only a single hotspot is observed, which we assign to single current filaments from which heat spreads into the top electrode. In addition, the position of the hotspot does not appear to vary during the course of our measurement series, i.e., operation over several hours. An important feedback possible due to the high resolution is that the position of the filament is not located at the device edges, where it might form due to compositional fluctuations and defects.

### 3.1 Hotspot Diameter

To discuss the diameter of the observed hotspot, an additional scan of only the hotspot was recorded for device 1 with a higher lateral resolution of 5 nm, of which the temperature profiles through the center and at different offsets are shown in Figure 4d. The temperature reaches about 0.1 K in the center of the hotspot with a narrow concave region around the center followed by a broad decay up to a radius of at least 200 nm.

To find the radius of the underlying heat-source, the heat spreading in the top electrode can be described by spreading from an isothermal circular heat-source^25^ (for large top electrode thickness *t*_TiN_, see supplementary). Alternatively, the radius can be found by calculating the inflection point of the temperature profile, which was confirmed by finite element simulation to overestimate the radius by only ∼10 nm in the system at hand. The extracted heat-source radius using the shape of the temperature decay is *r*_f_ ≈ 100–133 nm. We note here that the extracted quantity is the heat-source radius, and not the filament radius. Still, this appears large compared to the expected diameter of the conductive filament of nanoscale down to molecular dimension. However, the exact biasing procedure and material stack can influence the size.^2^ In addition, heat spreading takes place inside the HfO_2_ layer, which could lead to further broadening of the effectively seen heat source. The heat-spreading could even lower the electrical conductivity in its surrounding, also leading to a larger heat-source diameter.^26^

### 3.2 Hotspot Temperature

Turning to the magnitude of the temperature rise, we note that the temperature rise is much smaller than the expected temperature of the current filament during switching on the order of hundreds of Kelvin. As already discussed in previous work,^4−6^ one explanation is the large thermal conductivity and heat-spreading in the metal top electrode. However, we find from finite-element simulations of the top electrode only, that the maximum temperature reached at the surface is very similar to the temperature at the electrode side of the material interface. Moreover, as pointed out by Deshmukh et al.,^4^ even more important is the thermal boundary resistance between the oxide and the metal electrode. At this point it may be most instructive to discuss their influence using approximate analytical equations that show the expected scaling with dimension. A simplified thermal circuit is depicted in Figure 4f. Starting with the thermal boundary resistance^27^ between TiN and HfO_2_
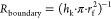
18we use *h*_k_ = 1 MW · m^–2^ K^–1^, and obtain about *R*_boundary_ ≈ 3.2 · 10^7^ K/W. Next, the heat spreads into the top electrode, which for *t*_TiN_ ≫ *r*_f_ one may approximate by
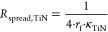
19

Using a value of κ_TiN_ ≈ 11 W/(m K)^28^ we obtain *R*_spread,TiN_ ≈ 2.2 · 10^5^ K/W. However, in the case at hand the filament diameter is larger than the thickness of the film. Therefore, we use a finite element simulation to calculate this resistance, resulting in *R*_spread,TiN_ = 1.7 · 10^6^ K/W, which is larger by 1 order of magnitude. Nevertheless, the boundary resistance is larger than the spreading resistance by 1 order of magnitude, with this ratio becoming even more pronounced if smaller filaments are expected. To understand the small magnitude of temperature drop at the electrode surface, we have to compare these two resistances to the further heat spreading inside the HfO_2_. The cylindrical heat spreading equation predicts a logarithmic scaling of thermal resistance with the size of the filament:
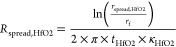
20with a thermal conductivity of HfO_2_ on the order of κ_HfO2_ ≈ 1 W/(mK), a thickness of *t*_HfO2_ = 4 nm and a decay to room temperature at radius *r*_spread,HfO2_ ≈ 100 nm, we obtain about *R*_spread,HfO2_ ≈ 2.6 × 10^5^ K/W. This is 2 orders of magnitude smaller than the thermal boundary resistance, and 1 order of magnitude smaller than the spreading resistance in the TiN layer. These conclusions are similar in terms of hotspot size and in support of previous findings.^4−6^ We note that our method can gain not only temperature but additional information from the phase and imaging thermal waves without the need for calibration.

The total thermal resistance and temperature are calculated to *R*_tot_ = 2.56 × 10^5^ K/W and, using the DC electrical power calculated above, Δ*T*_f_ = 18.2 K above room temperature. This is a plausible temperature since during our measurement, which is equivalent to a read operation, the filament is stable and does not switch.

The proposed approach could be applied to transient processes like set/reset operations, in principle, given the ratios of device temperature harmonics, β, can be determined from the ratio of power harmonics. This implicitly assumes that the thermal resistances within the device do not depend on the temperature in the range reached through this self-heating. Over a large temperature range, as expected for a full switching cycle of RRAM, this needs to be verified further, as the self-heating could significantly vary the conduction path and therefore the thermal resistance of the filament.^26^ Note, this method uses lock-in detection and therefore averages over many set/reset cycles, requiring a certain device stability.

### 3.3 Noise Discrimination

The method presented in this work provides further insight: there is additional useful information in the phase signal obtained in the phase-sensitive detection of the thermal signal using a lock-in amplifier. If there is a clear phase signal with a value indicative of phase-locking, then we can confidently analyze the results. In return, a noise-phase signal is indicative of a lack of phase-locking and we can conclude that the thermal amplitude signal is not related to the device heating by the applied AC current. This is an important and useful way to discriminate errors and confidently tell apart small signals from noise, which appears more difficult to do using conventional SThM techniques. The temperature signal in Figure 4 is plotted in such a way that only in the significant (phase locked) regions a temperature is indicated by the color bar, while other regions remain in the greyscale of the topography image. We found this a useful way to display data for various samples. More details and further noise considerations are found in the Supporting Information.

### 3.4 Observation of Diffusive Wave

The phase information has even more merits. We can identify regions in which the phase is locked to the electrical excitation at a different value from the one in the center of the hotspot. In such regions, a phase lag can be attributed to a time delay in heat spreading. In Figure 4e, we report the radial evolution of the phase signal away from the center. Three different regions can be identified. First, in a radius of up to 100 nm around the hotspot center, the phase is almost constant. In this region, the ratio of signal amplitudes is also constant and close to the expected ratio β_1_/β_2_ (see supplementary). This confirms the assumption of the temperature following the power without time delay, i.e., steady state operation. The region without phase locking is seen further out for radial distances larger than about 180 nm. As stated above, the associated small but still visible thermal amplitude signal needs to be handled with care.

In between these trivial regions, we observe an almost linear decrease of the signal with increasing radius. In this region, we attribute a phase lag to the finite thermal diffusivity. Importantly and excitingly we can access this observable not easily obtained for nanoscale materials. We intend to extract quantitative values of diffusivity from such data in a dedicated study. A phase deviation can generally be used to avoid errors in making a steady state assumption. We give an example in the Supporting Information.

### 3.5 Device Design Considerations

To motivate further study, we draw several preliminary conclusions from these observations.

According to our calculations, the total thermal resistance and temperature of the filament are dominated by the spreading inside the HfO_2_, which is made possible by the large thermal boundary resistance. Moreover, the small observable temperature at the top-electrode is dominated by the large thermal boundary resistance, which confines the heat to dissipate through the HfO_2_ layer. In combination with modeling, which takes the geometries into account, the SThM data feeds into thermal conductivity, thermal diffusivity and thermal boundary conductance values. This is important because these quantities are dependent on the deposition process and film thickness. Therefore, relying on literature values may lead to a significant systematic error in modeling.

## 4 Conclusions

In this paper, we have derived a method to obtain in-operando quantitative temperature information about nanoscale nonlinear devices. We verified the proposed approach by imaging a calibrated device of homogeneous temperature and found good agreement between the measured and expected temperatures, if the sensor’s temperature is well-known and calibrated. The used approach can be applied easily to a network of nonlinear devices if the β_1_ and β_2_ of the network memristive elements are equal; else it has to be extended further. We also show how the temperature rise due to Joule and Peltier heating can be distinguished using this method. The proposed method mitigates artifacts from tip–sample thermal resistance variations. Additionally, phase information can be used to identify regions where the calculated temperature and tip–sample thermal resistance are still erroneous, as is shown in images of RRAM devices.

The method was applied to HfO_2_-RRAM devices. The underlying switching mechanism in RRAM consists of movement of oxygen vacancies, which is not directly observable with nanoscale resolution. However, due to its direct dependence on temperature, conclusions about thermal transport are useful for device modeling and design. The low temperature values suggest that the thermal boundary resistance cannot be ignored in such devices, especially with smaller radii of heat-sources than found in our devices. Important information (effective heat-source radius) can be extracted from high-resolution temperature images, especially when other information is missing.

## 5 Methods

### 5.1 Derivation of Equations for Nonconstant Current

So far, we have derived eq 13 to calculate the sample’s temperature rise for the case of constant current in the sensor. This holds true as long as the resistance changes in the cantilever are negligibly small in comparison to the constant resistance in the Wheatstone-bridge. This can be enhanced, at the cost of lower sensibility to sensor-resistance changes, by choosing a series resistor in the Wheatstone-bridge which is much larger than the sensor’s resistance.

In the general case of *I* ≠ const., we need to rewrite eq 6 as
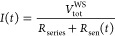
21which leads to the expression
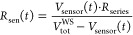
22which is not trivial to split into the different constant and frequency components due to *V*_sensor_(*t*) appearing in both the numerator and the denominator, but is necessary to obtain the sensor’s resistances Δ*R*_DC_, Δ*R*_AC,1ω_ and Δ*R*_AC,2ω_ with their corresponding temperature changes and the electrical power components *P*_el,DC_, Δ*P*_el,AC,1ω_ and Δ*P*_el,AC,2ω_.

We note that in the Wheatstone-bridge, when the cantilever’s resistance changes periodically at a frequency *f*_0_, the Wheatstone-bridge voltage will have a component at that frequency and, due to the nonconstant current, also components at higher harmonics of the frequency with decreasing amplitude. To describe the signal created in higher harmonics, we can write the Wheatstone-bridge voltage dependent on the (unknown) cantilever resistance change Δ*R*_AC,1ω_ at angular frequency ω_0_ = 2π *f*_0_ as
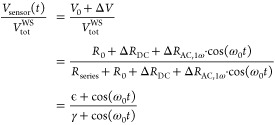
23where ϵ := (*R*_0_ + Δ*R*_DC_)/Δ*R*_AC,1ω_ and γ := (*R*_series_ + *R*_0_ + Δ*R*_DC_)/Δ*R*_AC,1ω_. Unfortunately, there exists no analytical solution for the Fourier transform of a signal of this form
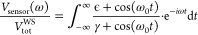
24

Instead, we obtain the coefficients of the Fourier series of *V*_sensor_(*t*) by means of comparison of coefficients:
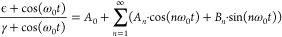

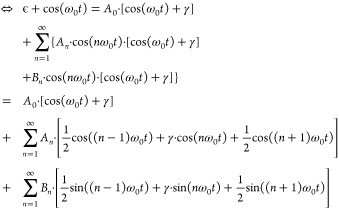


Comparing the coefficients, we see that all *B*_*n*_ = 0 and are left with *n* equations for *n* + 1 coefficients *A*_*n*_. Since we know that the *A*_*n*_ decrease monotonically, we can reduce the number of variables to *n* by setting *A*_∞_ = 0 and can solve for an expression for the *A*_*n*_ that only depends on the *x*_*n*_
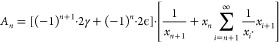
with the *x*_*n*_
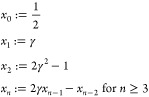
and find the ratio between the signal at harmonic *n* and the following harmonic *n* + 1
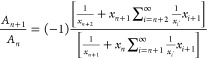
Using γ ≫ 1 for the typical values during the measurement, this finally yields the ratio
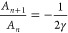
25

We can interpret the result as the amplitude of V becoming smaller with  while the alternating sign of the amplitude corresponds to a phase shift of π. In our case, γ is large, and when detecting the resistance change in 1ω it is sufficient to use the voltage signal detected in 1 ω, neglecting the 2ω signal. However, when sufficiently high 1ω resistance change is present, it creates a 2ω component in the voltage as well, so that the 2ω voltage signal can not readily be used to calculate the 2ω resistance change. In such cases, it might be beneficial to replace the voltage-source with a current source.

In the experiment, we can either use a Taylor-expansion, here to the second order, to calculate the sensor’s resistance
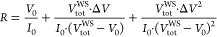
26or a Discrete-Fourier-Transform on the time dependent signal *R*_sen_(*t*). The electrical power in the heater can then be calculated and separated into three different contributions at 1ω, 2ω and DC:
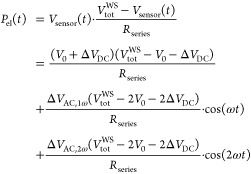
27using 5 and neglecting second order terms Δ*V*_AC,1ω_ · Δ*V*_AC,2ω_,  and . Now we can use the obtained Δ*R*_DC_, Δ*R*_AC_, *P*_el,DC_ and Δ*P*_el,AC_ together with the initial eqs 7 to 10 to solve for the desired sample temperature and tip–sample thermal resistance:
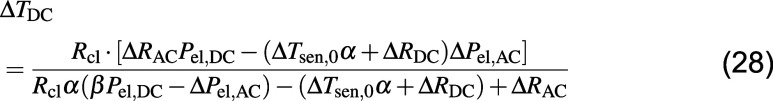
28and
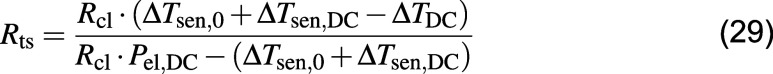
29

To ease reading, the results of 28 were not plugged into 29.

### 5.2 Combining Joule Heating with Peltier Effects

To take into account and quantify the Peltier effect relevant for some device types, the simultaneous measurement at different harmonics can be used. Because the Peltier cooling/heating scales with the current *I* while the Joule effect scales with power (or *I*^2^ for linear devices) the two effects appear differently at the different harmonics. In the case shown above, comparing the different harmonics confirms that Peltier effects can be neglected. However, to indicate how to proceed in other cases, we show the equations for a linear device below, which also shows how to extend the calculations to nonlinear cases.

In the case of a linear device that shows Joule heating and Peltier heating/cooling, the temperature rise in the device can be inferred as
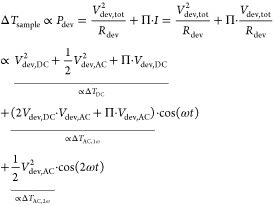
30where Π is the Peltier-coefficient. This takes into account the temperature change due to Peltier heating/cooling, but does not correspond to the local thermovoltage directly due to heat propagation in the device. The definitions of β_1_ and β_2_ are used according to 16 and 17, as before, only for the Joule heating temperature rises. Then the initial eqs 7 to 10 are used again and separated into their constant, 1ω- and 2ω-components. We obtain three equations which implicitly contain the variables Δ*T*_DC_, *R*_ts_ and Π such that all equations have to be used in the case at hand. Using the frequency components of the sensor’s resistance Δ*R*_DC_, Δ*R*_AC,1ω_, Δ*R*_AC,2ω_ and electrical power *P*_el,DC_, Δ*P*_el,AC,1ω_, Δ*P*_el,AC,2ω_ obtained with the constant current or alternative method as described above, the sample temperature can be calculated:
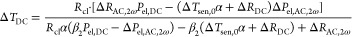
31

We note that the 2ω-component must be used in his case and is not interchangeable with the 1ω-component. The tip–sample resistance can be calculated using the previously derived eq 29, again not plugging in 31 for readability. The Peltier-coefficient is calculated:
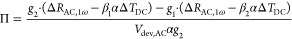
32with *g*_1_ = Δ*P*_el,AC,1ω_ α *R*_cl_ – Δ*R*_AC,1ω_ and *g*_2_ = Δ*P*_el,AC,2ω_ α *R*_cl_ – Δ*R*_AC,2ω_. The constant and first harmonic temperature rises in the device due to the Peltier effect can then be calculated from
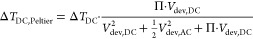
33
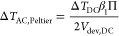
34and the temperature rise due to Joule heating can be found using 16 and 17.

## Supporting Information Available

Description of the experimental setup, details of the metal thin film heater which was used as the calibration sample, a description of the influence of laser heating on the cantilever and the energy equation, artifact detection in using the obtained phase-signal, a detailed description of the filament width estimation, and uncertainty considerations (PDF)

## Notes

^†^ Max Planck Institute for the Structure and Dynamics of Matter, 22761 Hamburg, Germany.
