# Supplementary material for: Scanning Thermal Microscopy Method for Self-Heating in Nonlinear Devices and Application to Filamentary Resistive Random-Access Memory
Source: ACS Nano. 2025 Jan 29;19(5):5342–52. doi: 10.1021/acsnano.4c12784 (PMC11823610; doi:10.1021/acsnano.4c12784)
Supplement: Supplementary file 1 — nn4c12784_si_001.pdf [file nn4c12784_si_001.pdf]

Scanning Thermal Microscopy method for self-heating in non-linear devices and application to filamentary resistive Random-Access Memory - SUPPLEMENTARY INFORMATION

Nele Harnack,[†] Sophie Rodehutskors,[†,‡] and Bernd Gotsmann[*,†]

†*IBM Research Europe - Zurich, 8803 Rüschlikon, Switzerland*

‡*Current address: Max Planck Institute for the Structure and Dynamics of Matter, 22761 Hamburg, Germany*

E-mail: bgo@zurich.ibm.com

26th September 2024

Experimental Setup

The Scanning Thermal Microscopy was conducted in the setup at the BRNC noise free labs in Zurich, Switzerland. The labs provide shielding against electromagnetic, acoustic and vibrational disturbances and are temperature and humidity stabilised, which is described further in Lörtscher et al.[1] The home built microscope described in more detail in Menges et al.[2] is set up in a high vacuum chamber at a pressure of 10^{-7} mbar and at a temperature of 21 °C. The thermoresistive probe utilising a doped Si-heater used here is biased in a Wheatstone-bridge with 2.5 V, which

conforms to $P = 1.3\,\mathrm{mW}$. It is calibrated prior to the measurement and consists of the sensor temperature at the working point $T_{\mathrm{sen},0}$ and its change of resistance with temperature change α, where $\Delta T_{\mathrm{sen},0}$ contributes linearly to the measured temperature as can be seen from (13). The full procedure is described in Spieser et al.[3] We note here that the calibration in this approach does not include the tip-sample thermal resistance, but solely describes the probe: the thermal resistance of the cantilever legs, R_{cl}, and the temperature of the sensor out of contact with the sample, $T_{\mathrm{sen},0}$.

To conduct the SThM measurements of the HfO_2 devices, the chip with the devices was glued to a printed circuit board using silver paint, which at the same time provides an electrical contact for the bottom electrode. The top electrode contact was wire bonded with $25\,\mu\mathrm{m}$ Al wire in a Devoltek wire bonder. The devices show binary switching between a high and a low resistive state HRS and LRS.

Details of the metal thin film heater (calibration sample)

Due to the very high aspect ratio and high thermal conductivity within the metal heater device, the resulting temperature distribution is expected to be homogeneous along its length, making the length to width ratio the most relevant design consideration. The device is shown in figure 2 g. As a result, a small part of the total surface area, such as in a scan, is representative of the overall temperature. Moreover, the average device temperature can be determined through measuring its resistance change in 4-probe configuration as a function of the applied current.

To calibrate the sample, it was placed in the temperature-controlled environment of a DynaCool PPMS. By applying a temperature ramp and reading out the 4P-resistance with an AC voltage of $50\,\mathrm{mV}$, a voltage small enough not to cause self-heating, the resistance-temperature relation of the device was measured.

Laser heating in cantilever

The cantilever deflection which is used for the AFM-capability of SThM is measured via the displacement of the laser dot reflected from the cantilever's top, as is typically done in AFM systems. In this case we also need to take into account that the light is not fully reflected from the cantilever. We need to consider that P_{el} is not the only 'input' heat flow during the calibration of the sensor both during the calibration and the measurement by replacing P_{el} with $P_{\mathrm{el}} + P_{\mathrm{laser}}$ in the energy equation. For calibration of the cantilever, we can see a shift in power between the R-P curve taken with or without the laser focused on the cantilever. By calculating this shift, we can determine the laser heating. During the measurement, we can replace the DC-energy equation by $P_{\mathrm{el}} + P_{\mathrm{laser}} = \dot{Q}_{\mathrm{cl,DC}} + \dot{Q}_{\mathrm{ts,DC}}$ while the AC-energy equation stays unaffected. In our setup typical values for the contributions of laser heating are $2.8 \cdot 10^{-5}\,\mathrm{W}$, i.e. $2\,\%$ of the heating power.

Phase for artifact detection and full data set: resistive RAM

In the main manuscript, we explain the usefulness of detecting the phase relationship of the thermal signal with respect to the applied device voltage. Here we give two examples, showing how the phase signal is used to verify that the assumptions of local equilibrium and no interference from the measurement process are valid. In addition, we give more consideration to the thermal wave interpretation.

In figure S1 the full dataset of the scan of device 1 is shown, from which the line scans in figure 4 d were extracted. The hotspot region is visible in both amplitude (region of largest amplitude) and phase signals (region with constant phase), where each signal decreases monotonically with increasing radial distance from the center of the hotspot. The amplitude of the temperature decreases due to heat spreading, while the decrease in phase can be explained by the presence of thermal waves. The presence of thermal waves can be confirmed by taking the ratio of the values of $\Delta V_{\mathrm{AC},1\omega}$ and $\Delta V_{\mathrm{AC},2\omega}$ as depicted in figure S3. They are consistent with the ratio of the predicted β_1 and β_2: $\Delta V_{\mathrm{AC},1\omega}/\Delta V_{\mathrm{AC},2\omega} \approx 3.4$ and $\beta_1/\beta_2 \approx 3.7$ in the center of the scan. They increase with

increasing radial distance due to the frequency dependent faster decay of the 2ω amplitude. Even further outside the hotspot region, the amplitude ratio seems to decrease again, which can clearly be attributed to the influence of the white phase noise when the signal gets too small.

In the topography images, several protrusions are visible which consist of residues from clean-room fabrication. Two of these lie in the region of the hotspot. At the protrusions, the amplitudes $\Delta V_{\mathrm{AC},1\omega}$ and $\Delta V_{\mathrm{AC},2\omega}$ are low and the phase signals are delayed which is consistent with contamination by particles of low thermal conductivity or poor thermal coupling to the electrode surface. The calculated thermal resistance signal R_{ts} and temperature rises ΔT_{DC} and $\Delta T_{\mathrm{AC},2\omega}$ are shown in figure S2 a-c. The calculated R_{ts} spans one order of magnitude from $1.2 \cdot 10^7\,\mathrm{K/W}$ to $1.1 \cdot 10^8\,\mathrm{K/W}$, which illustrates the general challenge of nanoscale thermometry: the large contrast in thermal resistance.

The second particle, which is indicated in figure S2 a, shows in an exemplary way that the method described here can correct for strong variations in thermal resistance. Despite the low thermal conductance of the particle, the particle is hardly visible in the extracted temperature map, in agreement with the expectation that the passive particle adopts the temperature of the underlying device region. This also indicates clearly that a change of sample properties through interaction with a hot probing tip is not significant. As an experimental indication, the clean phase signal in the particle area (figure S1 e and f) is a good measure, only showing deviations at the edges, where the signal changes too suddenly for the measurement bandwidth.

Turning to the first particle, we present an example where the analysis assumptions do no longer hold. The tip-sample thermal resistance is particularly large in this area. In this case, the calculated temperature rises ΔT_{DC} and $\Delta T_{\mathrm{AC},2\omega}$ are much larger than those in the underlying hotspot region. In the Θ_2 phase-signal, this artifact can be clearly identified: it has a distinctly different phase from the center region of the hotspot, and does not show the same decline of the phase as the surrounding of the hotspot does.

Consequently, we can use the phase information to create a confidence map and separate the erroneous region. To do so, the phase of the center of the hotspot of 1.61π is chosen as the trusted

phase, and a phase deviation of 0.01π is chosen as a threshold, as shown in figure S2 f. Then, a 3x3 filter is applied to each pixel, where each pixel is decided to be trustworthy if at least 5 of the pixels in the grid are within the trusted phase interval, creating a confidence map as depicted in subfigure d. This additionally robustly removes pixels in which the amplitude signal is approaching the noise level, such that the temperature can not be calculated correctly. The resulting trusted region of the DC temperature rise is shown in subfigure e, where the removed artifact is indicated by a pink circle. The remaining trusted area consists of the hotspot and few, small areas throughout the scan with a locked phase. Since the choice of narrow phase-filtering has cut out the radial heat spreading in the surrounding of the hotspot, the data depicted in the main text in figure 4 d and e contains all vertical line profiles to the left of the hotspot center instead of the confidence area. Regardless, we stress the possibility of using the phase signal for detecting not-trustworthy areas, resulting from both artifacts and regions of signal close to the noise floor, and filtering them out.

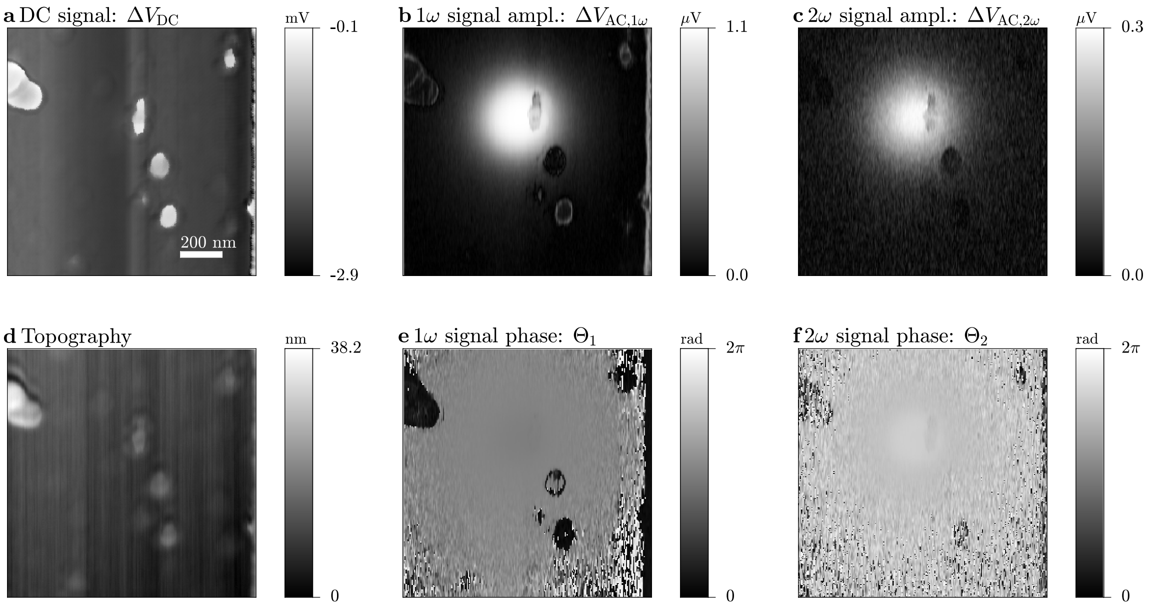

Figure S1: High resolution (5 nm) image of hotspot in device 1. DC voltage change relative to out-of-contact value (a) and AC voltage amplitude in 1ω (b) and 2ω (c). Topography of device (d) and AC voltage phases in 1ω (e) and 2ω (f). The 1ω signal is corrected for crosstalk: the amplitude ΔV_{cross} and phase Θ_{cross} of electrical crosstalk are extracted from 30x30 pixels in the bottom left corner of the raw signal. The 1ω signal is then corrected to the complex $\Delta V_{\text{AC},1\omega,\text{C}} \cdot \exp^{i\cdot\Theta_{1,\text{C}}} - \Delta V_{\text{cross}} \cdot \exp^{i\cdot\Theta_{\text{cross}}}$, of which the amplitude and phase are plotted in (b) and (e).

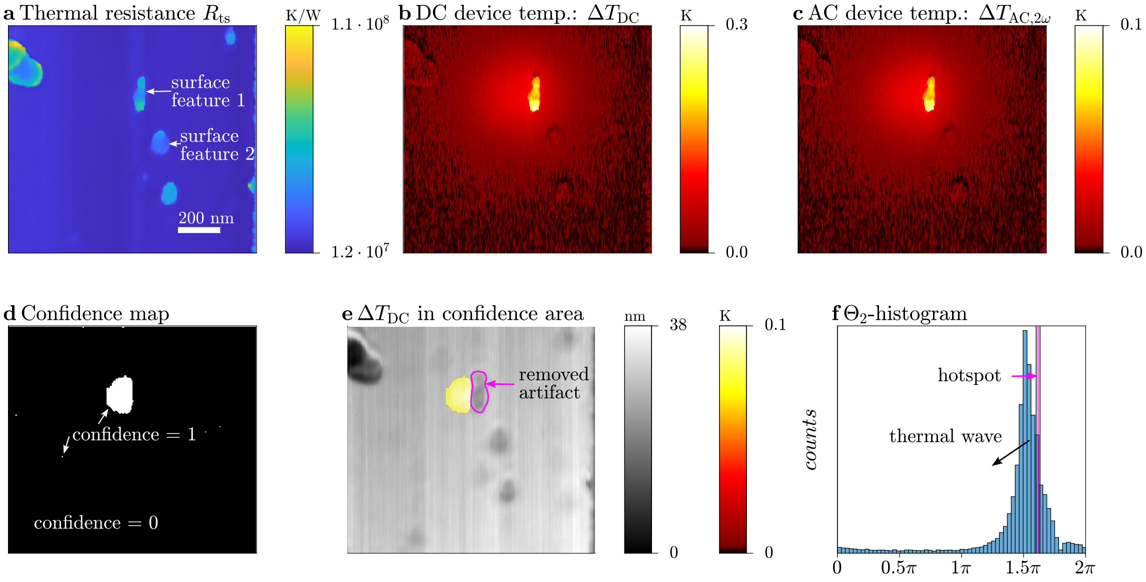

Figure S2: Tip-sample thermal resistance R_{ts} (a), DC and AC temperature rise above room temperature in K (b,c). Confidence map, where 0 is equivalent to not trustworthy, and 1 is equivalent to trustworthy (d). DC temperature rise in the confidence area, in overlay with topography (e). Phase-histogram of Θ_2 indicating the trusted interval of phases and the decay due to thermal waves

Estimation of filament width

Due to the filament being buried beneath the thermally well conducting top electrode, the properties of the filament are not directly accessible. However, making use of a simplified, analytical model of the heat-flow within RRAM cells and comparing the result with the measured temperature distribution, some boundaries to the parameters of the system can be identified.

According to Yovanovich and Marotta,[4] the temperature distribution at a given height z and radial distance r from an isothermal, circular heat source in a semi-infinite half-space has an analytical solution and can be described as follows

$$T_{\mathrm{TE}}(r,z) = \frac{2}{\pi} T_{\mathrm{TE},1} \cdot \sin^{-1}\left(\frac{2 \cdot a}{\sqrt{(r-a)^2+z^2} + \sqrt{(r+a)^2+z^2}} \right) \tag{S1}$$

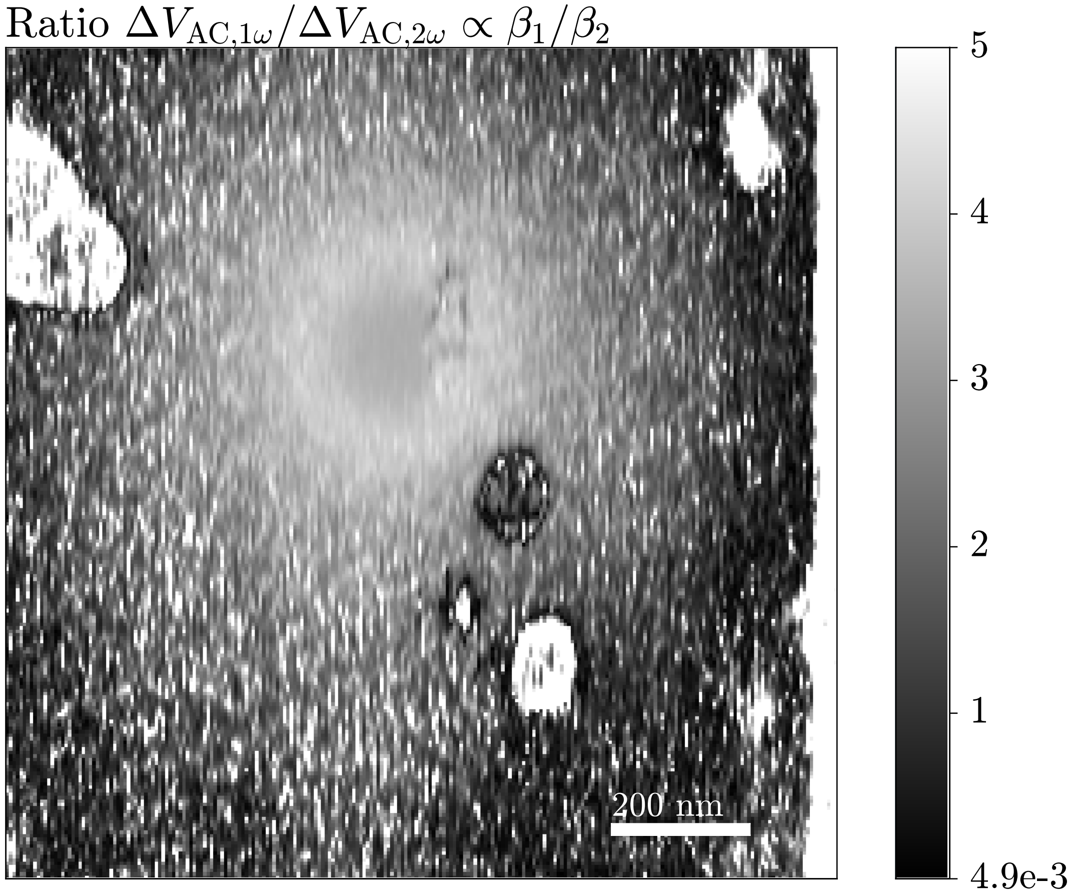

Figure S3: Ratio of the amplitude signals $\Delta V_{\mathrm{AC},1\omega}/\Delta V_{\mathrm{AC},2\omega}$ with measured central value of 3.4, which is slightly smaller but consistent with the expected value β_1/β_2=3.7. It increases with the radial distance, indicative of amplitude decay in the thermal wave.

The calculated distribution depends only on geometrical factors, but not on material parameters: the radius of the heat-source, a, and the temperature of the heat-source, $T_{\mathrm{TE},1}$. Assuming that the temperature distribution on top of the finite electrode can be described well by the temperature distribution according to (S1) at the height of the top electrode thickness, one can use the analytical solution to extract the radius of the filament. That this approximation gives a reasonable indication is easily verified using finite element modeling.

The experimental temperature data is shown in figure S4. The central line profile along the fast scan axis through the hotspot is indicated in red. The orange lines indicate the two neighbouring temperature profiles. The black solid lines indicate the expected temperature distributions for assumed filament diameters from $0.1\,\mathrm{nm} - 1\,\mathrm{nm}$ and in logarithmic spacing from $10\,\mathrm{nm} - 1\,\mu\mathrm{m}$. The two thick black lines give the best fit to the overall shape of the central profile, which yields a heat-source radius between $100\,\mathrm{nm}$ and $133\,\mathrm{nm}$. We note, however, that around the center the experimental temperature profile is less flat than predicted. This could be indicative of a limitation of such modelling describing the filament itself in a simplified manner. However, even when taking into account other filament models, the heat-source radius extracted here is either extracted correctly or overestimated, such that the extracted value gives an upper boundary of the heat-source radius in the studied RRAM cell.

Uncertainty considerations

Most of the uncertainty calculations and estimations made previously using the dynamic SThM method using similar cantilevers and the same setup still apply to this case. Previously it was found that the largest systematic uncertainty is related to the calibration of the sensor temperature, which was estimated to be in the range of 20 to 30%.[2] Using the verification measurements on the metal heater presented here, we estimate this uncertainty to be correctable. The measurement sensitivity if often estimated only based on the electrical noise of the sensor and would be $< 1\,\mathrm{mK}$ in our setup. We believe it is more relevant to extract it from a measurement including all aspects,

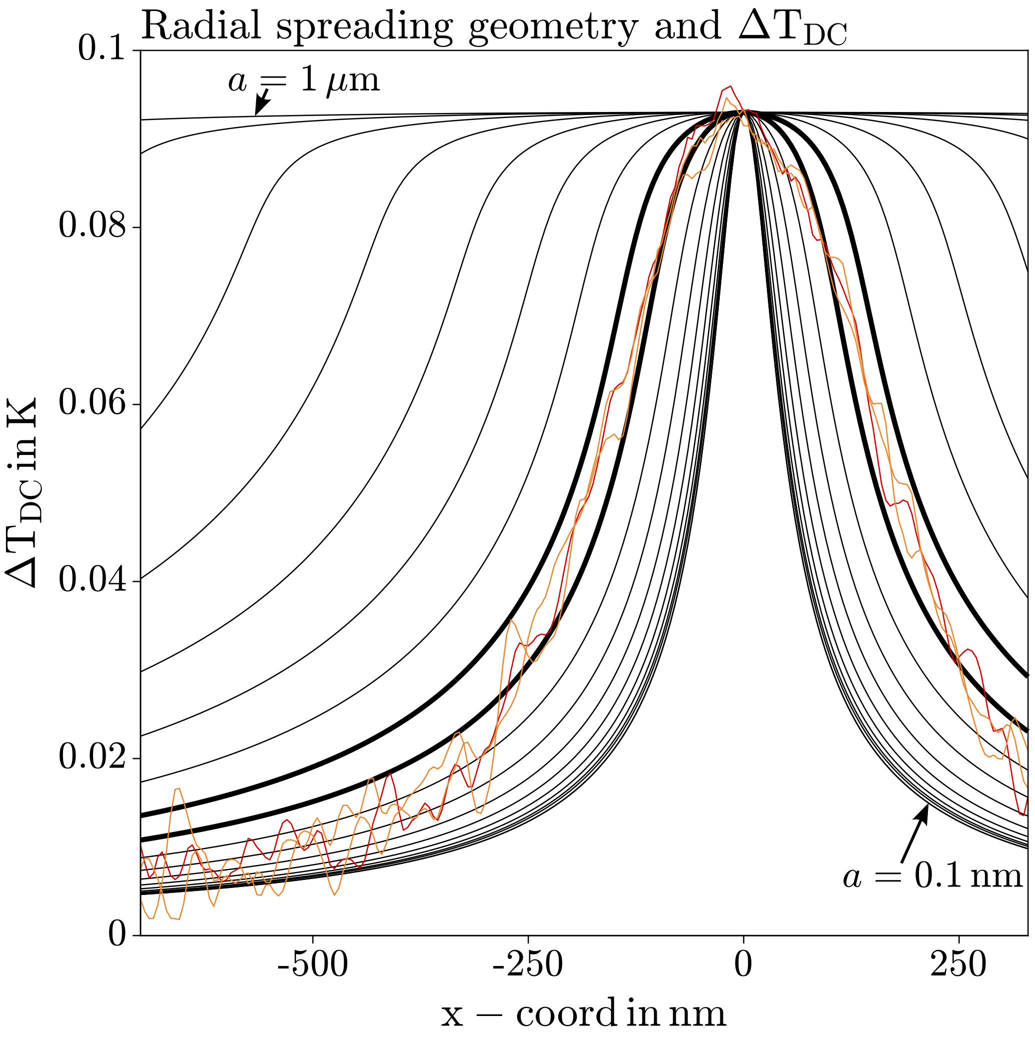

Figure S4: Temperature profiles in radial spreading geometry with isothermal circular heat source of radius a according to (S1) with radii of 0.1 nm and 1 nm, then in finer logarithmic spacing from 10 nm to 1 μm in black, thickened for 100 nm and 133 nm. Measured temperature profile through the center of the hotspot in red and the neighbouring 2 profiles in orange.

i.e. use the noise visible in calculated pictures of the extracted temperature. The noise extracted depends on the measurement parameters including the chosen bandwidth, the specific sensor used, and lock-in settings. For example using the line scans of figure S4 in the region outside the hot spot, we estimate 5 mK noise, and a larger value for the measurement shown in figure 2.

Apart from the measurement uncertainties, the fluctuations in the temperature of the self-heated

device during operation may lead to noise and uncertainty. On the scale of the pixel-time these would be averaged using lock-in detection. From our measurements of electrical device properties we further conclude that such fluctuations are negligible compared to the uncertainty and noise of the SThM measurement. For the small temperature regime studied here, we do not expect large fluctuations in the filament (e.g. due to oxygen vacancy movement)[5] and therefore do not expect large fluctuations in power dissipation.

References

(1) Lörtscher, E.; Widmer, D.; Gotsmann, B. Next-generation nanotechnology laboratories with simultaneous reduction of all relevant disturbances. *Nanoscale* **2013**, *5*, 10542–10549.

(2) Menges, F.; Riel, H.; Stemmer, A.; Gotsmann, B. Nanoscale thermometry by scanning thermal microscopy. *Review of Scientific Instruments* **2016**, *87*, 074902.

(3) Spieser, M.; Rawlings, C.; Lörtscher, E.; Duerig, U.; Knoll, A. W. Comprehensive modeling of Joule heated cantilever probes. *Journal of Applied Physics* **2017**, *121*, 174503.

(4) Yovanovich, M. M.; Marotta, E. E. Thermal Spreading and Contact Resistances. *5th International Topical Meeting on Nuclear Plant Instrumentation Controls, and Human Machine Interface Technology (NPIC and HMIT 2006)* **2006**, *2006*, 828–835.

(5) Menzel, S.; Waters, M.; Marchewka, A.; Böttger, U.; Dittmann, R.; Waser, R. Origin of the ultra-nonlinear switching kinetics in oxide-based resistive switches. *Advanced Functional Materials* **2011**, *21*, 4487–4492.
